# Live cell imaging of DNA and RNA with fluorescent signal amplification and background reduction techniques

**DOI:** 10.3389/fcell.2023.1216232

**Published:** 2023-06-05

**Authors:** Song Lu, Yu Hou, Xian-En Zhang, Yunhua Gao

**Affiliations:** ^1^ Center for Advanced Measurement Science, National Institute of Metrology, Beijing, China; ^2^ Institute of Synthetic Biology, Shenzhen Institute of Advanced Technology, Chinese Academy of Sciences, Shenzhen, China; ^3^ Faculty of Synthetic Biology, Shenzhen Institute of Advanced Technology, Shenzhen, China; ^4^ National Laboratory of Biomacromolecules, Institute of Biophysics, Chinese Academy of Sciences, Beijing, China

**Keywords:** live cell imaging, DNA and RNA dynamics, signal amplification, fluorescent background reduction, microscopy

## Abstract

Illuminating DNA and RNA dynamics in live cell can elucidate their life cycle and related biochemical activities. Various protocols have been developed for labeling the regions of interest in DNA and RNA molecules with different types of fluorescent probes. For example, CRISPR-based techniques have been extensively used for imaging genomic loci. However, some DNA and RNA molecules can still be difficult to tag and observe dynamically, such as genomic loci in non-repetitive regions. In this review, we will discuss the toolbox of techniques and methodologies that have been developed for imaging DNA and RNA. We will also introduce optimized systems that provide enhanced signal intensity or low background fluorescence for those difficult-to-tag molecules. These strategies can provide new insights for researchers when designing and using techniques to visualize DNA or RNA molecules.

## 1 Introduction

The spatial-temporal dynamics of DNA and RNA within living cells play critical roles in biological function, such as DNA replication and RNA transport (Mor et al., 2010; Zhou et al., 2017; Dovrat et al., 2018; Tsirkas et al., 2022). The advancement of microscopy techniques has led to significant progress in the observation of dynamic DNA and RNA behaviors (Shao et al., 2018a; Li et al., 2019). Fluorescent labeling techniques are also essential for accurately recording the behavior of nucleic acids. The signal-to-noise ratio (SNR) or signal-to-background ratio (S/B ratio) can be used to assess the imaging quality using various labeling techniques (Sandison and Webb, 1994). The noise in the SNR is represented by the standard deviation of the background fluorescence signal (Yang et al., 2019), whereas the background in the S/B ratio is defined by the average background fluorescence intensity (Tasan et al., 2020). Therefore, amplifying the signal or reducing the fluorescent background can achieve a higher SNR and S/B ratio, resulting in more efficient and effective labeling systems. Herein, we will focus on techniques for fluorescent tagging of DNA and RNA in live cells, as well as methods for signal amplification and background reduction, which provide useful tools for efficient imaging. Additionally, we will discuss several applications of these labeling systems.

## 2 Real-time DNA imaging tools in live cells

### 2.1 LacO/LacR and TetO/TetR

**FIGURE 1:**
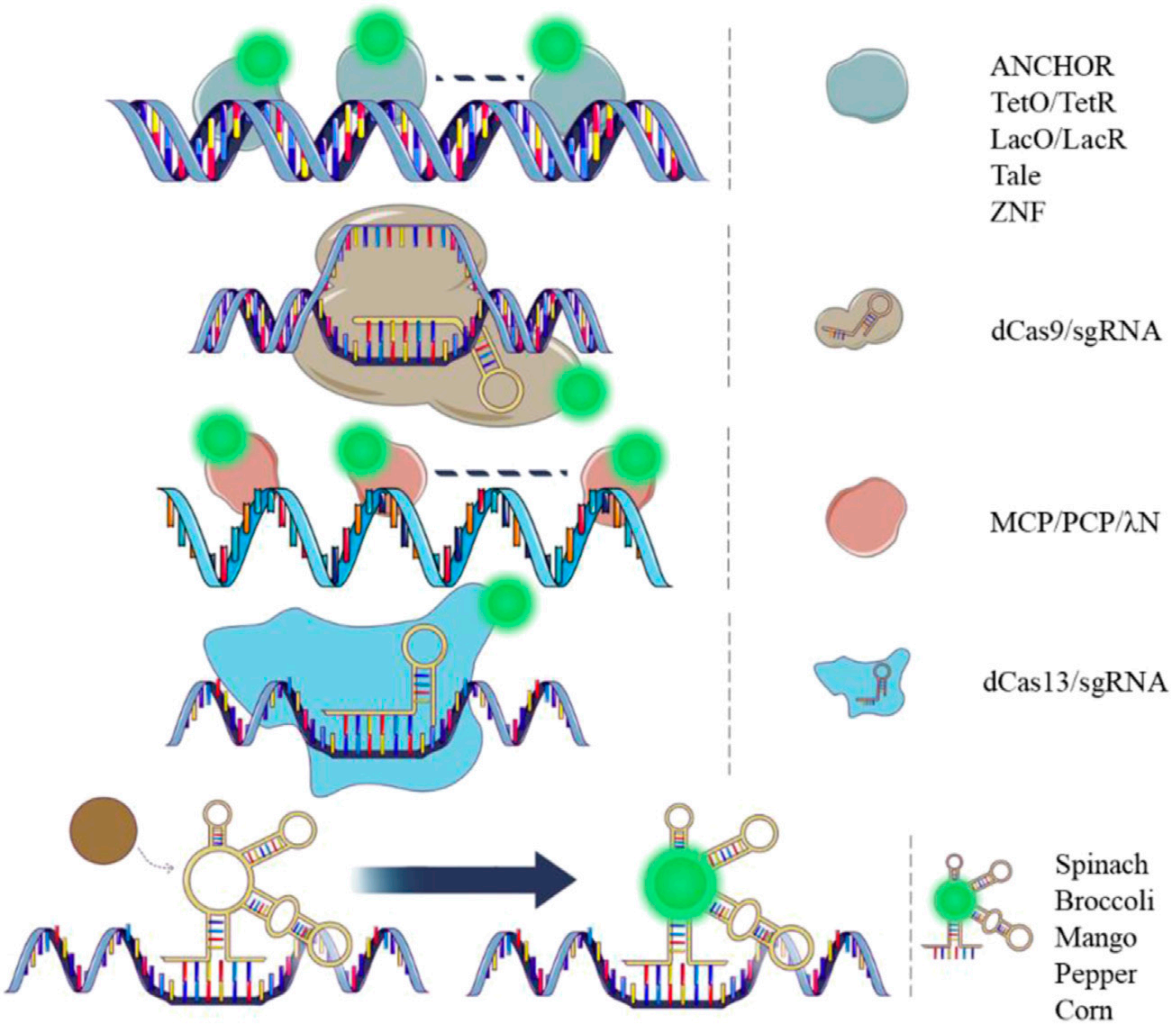
Dynamic DNA and RNA imaging tools. Images showing DNA (double strand) and RNA (single strand) tagging tools with different principles. Green spots represent fluorescent protein or fluorophore.

The principles of *in vivo* DNA labeling technologies involve the recognition of proteins that bind to specific DNA sequences directly or indirectly (as shown in Figure 1). Binding specificity and fluorescent brightness on the target genomic locus are essential factors in this process. In 1996, the Lac Operator-Repressor system (LacO/LacR) was first constructed to study *in situ* chromatin dynamics in live human cells (Robinett et al., 1996). This system involved the generation of 256 lac operators that were inserted into a mammalian DHFR (dihydrofolate reductase) expression vector. A green fluorescent protein (GFP) and a nuclear localization sequence (NLS) were fused to the lac repressor. The GFP-lac repressor-NLS fusion protein was then designed to bind the lac operator repeat *in vivo*. This system enabled dynamic visualization of a single copy plasmid and specific chromatin regions in live cells using light microscopy. It provided a new tool for *in vivo* dynamic studies of the genome and a direct comparison with fluorescence *in situ* hybridization (FISH) results. One year later, the Tetracycline Operator/Repressor system (TetO/TetR) was introduced. This system involved the integration of 336 Tet operators into a yeast chromosome, and a Tet repressor fused to GFP was expressed. The system was used to study sister chromatid cohesion in live yeast cells (Michaelis et al., 1997). To achieve a high enough fluorescent signal for the target of interest over the background fluorescence, both of the systems integrated long DNA arrays into the chromosome (more than 10 kb) to recruit hundreds of fluorescent proteins. However, such integration of long DNA arrays might affect gene function and induce recombination (Jacome and Fernandez-Capetillo, 2011; Lucas et al., 2014). Reducing the copy numbers of the operator array will alleviate the side effect on the gene function (Jovtchev et al., 2008; Tasan et al., 2018). Another optimization strategy is to mutate the TetR protein to alter its binding affinity for TetO (Krueger et al., 2007). This could potentially reduce the repression of the transcription activity by the TetR/TetO system.

### 2.2 ANCHOR

In the genome of *Burkholderia cenocepacia*, ParB proteins expressed by the *parABS* can bind the ParB-binding (*parS*) centromere sites (Dubarry et al., 2006). The cluster of *parS* sites (<1 kb) can recruit multiple ParB proteins. There are two clusters of *parS* sites in *B. cenocepacia*, respectively *parS*c2 (*parS* on chromosome 2) and *parS*c3 (*parS* on chromosome 3). The segment of DNA containing a cluster *parS* of sites was then adapted for use in eukaryotes and renamed as “INT” (Saad et al., 2014). The *parS*c2 site was renamed as INT1 and *parS*c3 site was renamed as INT2. The ParB proteins recognize the INT segment and multimerize on this region (Guo et al., 2022). When fused with a fluorescent protein (ParB-FP), the ParB-FPs create fluorescent loci. The ParB proteins and INT segments were referred to as ANCHOR, which include ParB1-FP/INT1 and ParB2-FP/INT2 pairs (Bystricky, 2015). These two labeling pairs are canonical and can be utilized simultaneously for dual-color labeling. The DNA double-strand breaks (DSB) were directly visualized by ANCHOR in living S. *cerevisiae*. The multimerization of 100–200 ParB proteins enabled dynamic recording of the resection process. An optimized ANCHOR/ParB DNA-labeling system was then developed for real-time imaging of a single-copy gene in human cells (Germier et al., 2017). The ANCHOR system’s ability for dual-color imaging and non-repetitive locus labeling makes it highly promising for more detailed visualization of the 3D genome. Furthermore, the ANCHOR system has been proved to be non-intrusive, as no create fragile sites were created by the insertions of INT.

### 2.3 ZF and TALE

Zinc finger (ZF) domains recognize specific DNA sequences consisting of 3 base pair triplets, which can be constructed to target a specific 9-bp sequence using a 3 ZF domain (Lindhout et al., 2007). This 3 ZF domain can then be fused with a fluorescent protein (ZF-FP) to label chromosomal loci containing copies of the 9-bp sequence. The major satellite repeat in mouse cells was labelled successfully by the system. However, the design of a new ZF for labeling a new sequence using this system can be laborious, as it relies on the recognition of specific triplet nucleotides (3-bp triplet). For instance, specific ZFs can recognize many of the 16 possible GNN triplets, but each ZF requires a separate plasmid construction (Segal et al., 1999). For recognizing other types of triplets, another family of ZFs should be selected by phage display. To address this limitation, the transcription activator-like effector (TALE)-mediated chromosomal tagging technique was developed to image highly repetitive sequences (Ma et al., 2013; Miyanari et al., 2013). The TALE protein mediates sequence-specific DNA recognition using repeat units containing 33–35 amino acids. In each repeat units, two critical adjacent amino acids are variable and specify one target base. These two residues constitute the repeat variable di-residues (RVDs). The TALE-FP system, which fuses TALE proteins with fluorescent proteins, is more flexible than the ZF-FP system for target sequence recognition. This is because each RVD can bind to a unique target base. The TALE-FP system has been successfully used for labeling telomeres and pericentromeres in human cells. To verify the system, telomere-specific protein TRF2 and FISH probe were used to colocalize with telomeres and pericentromeres, respectively. However, it should be noted that the plasmid construction of the system is also laborious, as it relies on the engineering of a new TALE protein containing specific RVDs for a specific target sequence.

### 2.4 CRISPR/dCas9

The clustered regularly interspaced short palindromic repeats system (CRISPR) has been adapted for live cell imaging using a complex consisting of deactivated Cas9 (dCas9) and a single-guide RNA (sgRNA), to which fluorescent proteins or fluorophores can be added. The dCas9-EGFP/sgRNA was first used in 2013 to label genomic loci with different copy numbers of repeat sequences (Chen et al., 2013). The system effectively labeled the second exon region of the MUC4 gene containing more than 100 repeats with 3 spots observed in over 50% of HeLa cells counted. A non-repetitive locus on MUC4 was also successfully labeled with more than 26 sgRNAs by lentiviral transfection. The system was robust with successful examination of FISH and other colocalization fluorescent proteins. A protospacer adjacent motif (PAM) close to the DNA target sequence of the dCas9/sgRNA is necessary in the system, restricting its flexibility for genome labeling. However, the dCas9-EGFP/sgRNA can still be a promising tool for repetitive genomic loci imaging with the use of a convenient sequence-editable sgRNA. In contrast, LacO/LacR and TetO/TetR systems require the knock-in of the repetitive sequences into the genome in advance for labeling a target locus. The 3D genomic organization has been studied using the dCas9-EGFP/sgRNA system (Wang et al., 2018a; Geng and Pertsinidis, 2021). Observing cells at different stages of mitosis is also achievable with the usage of the technique (Chen et al., 2013; Stanyte et al., 2018). In 2015, orthogonal Cas9 variants from three bacterial species (*Streptococcus pyogenes* (Sp), *Neisseria meningitidis* (Nm), and *Streptococcus thermophilus* (St1)) fused to different colors of fluorescent proteins were utilized for multicolor labeling on genomic loci (Ma et al., 2015). Dual color labeling for a given locus with highly repetitive sequences was achieved by using two of the three orthogonal dCas9. This technique increases the labeling ability of the CRISPR/imaging system for more than 2 target sites with multiple colors. In 2016, another Cas9 variant derived from *Staphylococcus aureus* (dSaCas9) was constructed for labeling genomic loci (Chen et al., 2016). Together with SpdCas9, two genomic loci spaced by less than 300 kb were resolved by the two labeling systems with dual colors. The dSaCas9 has a shorter PAM sequence compared to dNmCas9 and dSt1Cas9, providing more potential target sequences for labeling. The CRISPR imaging systems discussed above can robustly image genomic loci with high repetitive sequences. However, to label genomic loci with low repetitive sequences, signal amplification and background reduction techniques need to be introduced, which we will discuss in the upcoming section.

## 3 RNA dynamic imaging tools

### 3.1 Techniques based on fluorescent protein

RNA tagging technologies in live cells can be divided into two categories: those based on fluorescent proteins and those based on chemical fluorophores (Figure 1). Fluorescent protein-based RNA imaging technologies have been developed for over two decades. These techniques have been extensively used for RNA visualization and functional studies. The MS2 labeling system was derived from RNA bacteriophages, in which the MS2 coat protein is a specific RNA binding protein. The MS2 coat protein (MCP) was fused to a fluorescent protein (MCP-FP) for illuminating RNA in this system (Peabody, 1993). MCP forms homodimers that bind to the MS2 binding sites (MS2) specifically with high binding affinity (Golmohammadi et al., 1993). In 1998, the system was first used to label *ASH1* 3′UTR (untranslated region) in living yeast. For the construction of the system, six MS2s were tandemly integrated into the mRNA and MCP was fused with GFP. (Bertrand et al., 1998). A nuclear localization sequence was engineered into the MCP-GFP to restrict the fluorescent proteins in the nucleus. The *ASH1* mRNA transport and localization was visualized by the MS2 system and validated by the FISH probes. Several years later, in live mammalian cells, 24 MS2s were introduced tandemly to the 3′UTR of β-*actin* mRNA, and an EGFP was fused to MCP with NLS_sv40_. Single molecule of β-*actin* mRNA was visualized by the system in living mammalian cells. Sufficient fluorescent proteins were recruited to the target RNA with an average of 33 copies per mRNA in the 24 MS2s system. This was the first time a single mRNA molecule in living mammalian cells was visualized. Since then, the system has been extensively used for live cell imaging of different types of RNA and has become a gold standard for RNA imaging. Ongoing improvements to this technique has enabled researchers to study the life cycle of RNA and investigate the bio-functional output of these molecules (Ha et al., 2018; Tutucci et al., 2018; Laprade et al., 2020). A most recent study improved the MS2 system by counteracting the effects of nonsense-mediated mRNA decay (NMD) pathway. The effect can be induced by the original MS2/MCP system, while the improved version adopted SUP35 or PAB1* (mutated PAB1) to the MCP-GFP to reduce the effect in yeast. In mammalian cells, the fusion proteins MCP-GFP-eRF3 or MCP-GFP-PABPC1* (mutated PABPC1) were generated to reduce the NMD (Li et al., 2022). In 2011, the PP7 system, which is similar to the MS2 system, was used to image nascent RNA in its transcription locus (Larson et al., 2011). The system is composed of a fluorescent protein fused to a PP7 coating protein (PCP-FP) and the PP7 binding site (PP7). PCPs also form homodimers to bind to PP7 with high affinity (Tars et al., 2000). Additionally, the MS2 and PP7 systems are orthogonal and can be used for dual-color imaging in live cells (Hocine et al., 2013; Horvathova et al., 2017). Another labeling system, called λN, was developed for assessing the localization, movement, and dynamics of RNA molecules in live cells (Daigle and Ellenberg, 2007). This system contains an arginine-rich peptide called λN_22_ derived from the phage λN protein and a unique minimal RNA motif called boxB (Austin et al., 2002). The binding affinity between λN_22_ and boxB is lower than that of the MS2 system. The λN system is also compatible with MS2 system for a dual-color imaging (Lange et al., 2008).

The fluorescent protein-based techniques described above are able to visualize RNA with exogenous tags. Endogenous RNA visualization mainly relies on CRISPR-based imaging systems. A dCas13/sgRNA complex has been adapted for live-cell RNA imaging (Yang et al., 2019). Eight catalytically dead Cas13 homologs fused with enhanced green fluorescent protein (dCas13-FP) from different sources were screened. The result shows that dPspCas13b is the most efficient dCas13 protein with the highest SNR for labeling RNA, followed by dPguCas13b. The length and sequence of the sgRNA in the dCas13-FP/sgRNA complex were then optimized. Paraspeckle dynamics were analyzed using this complex, revealing a “kiss and-run/fusion” mode in live cells. The system was compatible with the MS2_24×_/MCP-FP labeling system and can be used for dual-color imaging of one RNA locus. The SNR of the dCas13-FP/sgRNA was half that of MS2_24×_/MCP-FP. However, the dCas13-FP/sgRNA showed significantly higher labeling efficiency than the MS2_24×_/MCP-FP, which may be due to the low knock-in efficiency of the MS2 tag on the target locus. Though the dCas13-based technique has the limitation of labeling sensitivity, the system has great capability for imaging large numbers of endogenous RNAs in living cells. Techniques of signal amplification and background repression can be combined with the dCas13-based system to achieve markedly higher SNR when labeling RNA.

### 3.2 Techniques based on chemical fluorophore

The first fluorophore-based labeling system is Spinach, which is an RNA-fluorophore complex that resembles enhanced GFP (EGFP). This system uses an RNA aptamer to mimic the GFP structure to light up the fluorophore (Paige et al., 2011). Spinach emits green fluorescence and has brightness comparable to EGFP. The 5S RNA was successfully labeled by Spinach in live HEK293T cells. The system exhibits negligible photobleaching compared to EGFP, making it adaptable for long-term imaging. Two years later, Spinach2 was developed with markedly increased brightness in living cells compared to Spinach (Strack et al., 2013). This system has enhanced folding and thermo-stability, which improves the versatility for RNA labeling in living cells. Spinach2 successfully labeled CGG repeat-containing RNA, which cannot be illuminated by Spinach. In 2014, Broccoli was built for RNA labeling with a shorter length of 49 nt. An increased fluorescence was achieved relative to Spinach2 (Filonov et al., 2014). The Broccoli system does not require exogenous magnesium addition and is tRNA-independent, making it a more useful tool for *in vivo* imaging. Mango system was also built in the same year. This system was reported to have a significantly higher affinity for RNA than Spinach (Dolgosheina et al., 2014). *In vivo* imaging of RNA was successfully demonstrated in *C. elegans*, while RNA dynamics in mammalian cells were not displayed by this system. Several years later, a series of new Mangos were developed for imaging the subcellular localization of small non-coding RNAs in mammalian cells. Two of these systems (Mango II and Mango IV) showed brightness equal to or higher than EGFP (Autour et al., 2018). In 2017, the Corn system was developed for quantifying RNA transcription in live cells (Song et al., 2017). This system emits red fluorescence with an emission maximum of 545 nm. The Corn system has improved photostability compared to Broccoli and Spinach in mammalian cells. Recently, a series of fluorescent RNAs called Peppers were developed (Chen et al., 2019). These are bright and stable monomers with a broad range of emission maxima spanning from cyan to red. The RNA aptamer used in the Pepper system does not contain a G-quadruplex structure, making it an ideal candidate for tagging functional RNA in cells. The system successfully labeled 5S ribosomal RNA, 7SK small nuclear RNA, and U6 splicing RNA. RNA polymerase-II-dependent transcription of mRNAs that cannot be labeled by Broccoli and Corn was visualized by Pepper, indicating superior performance for mRNA labeling. When co-labeled with the classic MS2/MCP system on a dual-tag RNA, Pepper showed much higher signal/background contrast than the MS2/MCP system (Chen et al., 2019). Techniques based on chemical fluorophores also include two other principles. One involves an RNA aptamer that interacts with the fluorophore within a fluorophore-quencher pair. This RNA aptamer separates the pair by a stronger binding affinity with the fluorophore than that between the fluorophore-quencher (Murata et al., 2011; Sunbul and Jaschke, 2013; Sato et al., 2015). The SRB-2/TMR-DN system contains an RNA aptamer (SRB-2), tetramethyl rhodamine (TMR; fluorophore), and dinitroaniline (DN; quencher). This system was used to image ribosomal and messenger RNAs in 2018 (Sunbul and Jaschke, 2018). Two years later, a system called Coral was developed with the same principle. It was reported to have greater brightness compared to Broccoli, Corn, and Mango when imaging mRNA or 5S ribosomal RNA (Bouhedda et al., 2020). The other involves an RNA molecular beacon carrying a fluorophore and a quencher on each end. The RNA molecular beacon emits fluorescence when the stem loop in the middle interacts with a target sequence and separates the fluorophore-quencher pair (Nitin and Bao, 2008; Rhee et al., 2008; Chen et al., 2017). The system was used to label single β-*actin* mRNA in growing axons with highly inclined and laminated optical sheet microscopy, which shows a relatively high sensitivity (Turner-Bridger et al., 2018). Both of the two techniques are non-fluorescent without the target molecules and emit fluorescence when they interact with the target and separate the fluorophore-quencher, enabling imaging of RNA with low background fluorescence in live cells.

## 4 Fluorescent signal amplification techniques

**FIGURE 2:**
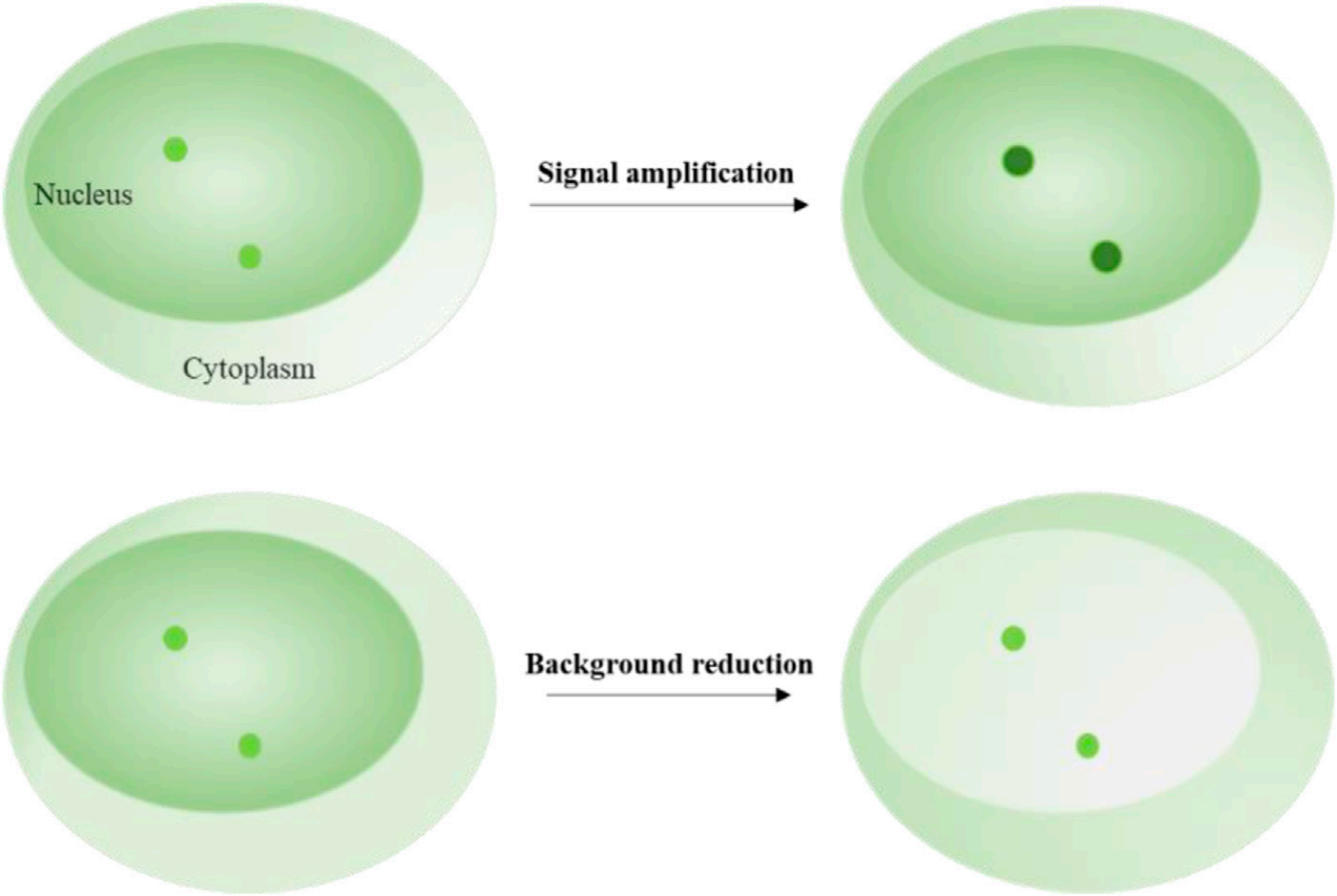
Schematics of signal amplification and background reduction. Upper image shows the fluorescent signal amplification strategy for labeling target loci. Lower image shows the fluorescent background reduction strategy for labeling target loci.

Signal amplification techniques are used to enhance the fluorescent intensity of target loci (Figure 2). These techniques typically rely on labeling elements that have been redesigned to include multiple fluorophores enrichment or a single fluorophore with enhanced brightness (Table 1). Signal enrichment is more commonly used to increase fluorescence intensity, possibly because the use of free single fluorophores with high brightness can lead to prominent background fluorescence readout. For example, a single quantum dot is usually bright enough to illuminate a locus of interest. However, labeling systems may introduce free quantum dots around the target signal, making it difficult to distinguish between the target and background signals.

**TABLE 1:** Toolbox with signal amplification and background reduction strategy.

Strategy	Representative labeling system	Performance	References
Multiple fluorophores enrichment			
Targeting highly repetitive sequence	ANCHOR	Non-repetitive locus containing “INT”	Saad et al. (2014), Germier et al. (2017)
	LacO/LacR; TetO/TetR	Non-repetitive locus containing >100 copies of operator sequence	Robinett et al. (1996), Michaelis et al. (1997)
	ZNF; TALE; dCas9/FP/sgRNA	Locus with >100 repeats^a^	Lindhout et al. (2007), Chen et al. (2013), Ma et al. (2013), Miyanari et al. (2013), Chen et al. (2016)
	MS2; PP7	Single RNA molecule	Hocine et al. (2013), Bauer et al. (2019)
Multiple fluorophores on one protein	dCas9-FP_3×_/sgRNA	Locus with >100 repeats	Ma et al. (2015)
	dCas9-SunTag/sgRNA	Locus with <100 repeats; Non-repetitive locus	Ye et al. (2017)
	dCas13-SunTag/sgRNA	Single RNA molecule	Chen et al. (2022)
Multiple fluorophores on one sgRNA	CRISPRainbow	Locus with >100 repeats	Ma et al. (2016)
	CRISPR-Sirius	Locus with <100 repeats	Ma et al. (2018)
	CRISPR FISHer	Locus with <100 repeats; Non-repetitive locus	Lyu et al. (2022)
	SIMBA	Locus with <100 repeats; Non-repetitive locus	Peng et al. (2022)
	dCas13/sgRNA/fluorogenic RNA array	Single RNA molecule	Tang et al. (2022)
Single fluorophore			
Quantum dots	TALE-QD	Non-repetitive locus	Ma et al. (2017a)
	dCas9-QD	Non-repetitive locus	Ma et al. (2017b)
Molecular Beacon	CRISPR/dual-FRET	Locus with <100 repeats; Non-repetitive locus	Mao et al. (2019)
Fluorescent intensity reduction			
BiFC	dCas9-SunTag/sgRNA/BiFC	Locus with <100 repeats	Hong et al. (2018)
	dCas13-SunTag/sgRNA/BiFC	Single RNA molecule	Chen et al. (2022)
	BiFC-TALE	Locus with <100 repeats	Hu et al. (2017)
Fluorogenic RNA	Spinach; Broccoli; Mango; Corn; Pepper	Multiple RNA molecules; Single RNA molecule	Paige et al. (2011), Dolgosheina et al. (2014), Filonov et al. (2014), Song et al. (2017), Chen et al. (2019), Cawte et al. (2020)
Number reduction			
	RNBS	Locus with <100 repeats	Lu et al. (2021)
	CRISPR-LIBR	Locus with <100 repeats	Hou et al. (2022)

^a^ Repeats represent repetitive sequences.

### 4.1 Multiple fluorophores enrichment strategy of labeling elements

To enrich more fluorophores on the locus of interest, one strategy is to target DNA or RNA loci with highly repetitive sequences using cognate fluorophore-tagging proteins. The highly repetitive sequence can be either endogenous or artificially engineered into the locus of interest. For the LacO/LacR and TetO/TetR systems, hundreds of tandem repeat sequences were engineered into the target region to recruit hundreds of fluorescent proteins, achieving higher signal fluorescent intensity than that of the background (He et al., 2000; Kanda et al., 2001; Cusanelli et al., 2013). TALE, ZNF, and CRISPR/dCas9 systems also adopted the same strategy to label highly repetitive sequences like telomeres and centromeres (Chen and Huang, 2014; Dreissig et al., 2017; Ren et al., 2017). In the MS2 and PP7 labeling systems, 24 copies of MS2 or PP7 are inserted into the target RNA to recruit up to 48 MCP-FPs or PCP-FPs, indicating that enough fluorescent proteins can light up a single molecule (Hocine et al., 2013). In another study, 128 copies of MS2 were integrated to enrich more fluorescent proteins, but the system did not exhibit a higher SNR in comparison with the 24-copy MS2 system (Dovrat et al., 2018; Bauer et al., 2019). This implies that increasing the SNR does not solely depend on the number of fluorescent proteins. As the number of target sequences available for binding to fluorescent proteins increases, the number of potential fluorescent proteins around the target locus will also increase, leading to increased background fluorescence. Among the fluorophore-based labeling methods, Mango II and Pepper aptamers were arrayed for RNA imaging in live cells with high contrast and achieved single-molecule sensitivity in several latest studies (Chen et al., 2019; Cawte et al., 2020). The systems showed marked improvements in signal to noise ratio in comparison with the MS2/MCP-FP labeling system, implying the potentially high sensitivity of these techniques. To image diverse endogenous mRNA species, a tandem array of inert Pepper (iPepper) fluorescence turn-on system was developed in live cells, with minimal disturbance to the target mRNAs (Wang et al., 2022).

Enriching more fluorophores on each protein or sgRNA that targets a specific sequence can reduce the number of sequences required to recruit enough fluorophores. This is another strategy that can be used to label low-repetitive sequences with high occurrence rates in the human genome. An example of this strategy is the dCas9/sgRNA/FP labeling system, in which three tandem fluorescent proteins have been fused to one dCas9 to increase signal intensity (Ma et al., 2015). The dCas9-Suntag system was then constructed, in which 10 or 24 fluorescent proteins can be recruited to one dCas9 (Tanenbaum et al., 2014; Ye et al., 2017). The dCas9-Suntag system has been used for low-repetitive and non-repetitive genomic loci, which increases the fluorescent signal-to-background ratio (S/B) in comparison with the dCas9-EGFP system (Shao et al., 2018b). Additionally, the dCas13-Suntag system has also been used to label RNA based on the same amplification principle (Xu et al., 2021; Chen et al., 2022). The sgRNA has been redesigned to insert two or more copies of MS2 or PP7 for MCP-FPs or PCP-FPs binding in CRISPR-based labeling systems (Fu et al., 2016; Shao et al., 2016; Wang et al., 2016). As a result, each dCas9/sgRNA/FP complex contains more than four copies of FPs, enhancing the brightness of each individual complex. Using this principle, a CRISPRainbow system was developed by fusing MS2, PP7, and boxB to stem loops or the 3′-end of the sgRNA, making it possible to image six chromosomal loci simultaneously (Ma et al., 2016). The redesigned system was also used for labeling repetitive sequence with fewer than 20 copies, known as CRISPR-Sirius (Ma et al., 2018). It contains octet arrays of aptamers in the sgRNA scaffold, enabling the complex to recruit up to 16 fluorescent proteins, leading to a higher S/B ratio compared to the complex with only two copies of aptamers. This optimized system provides a useful and robust tool for imaging more chromosomal loci. Another redesigned system integrates up to 16 MS2 binding motifs into the sgRNA to amplify fluorescence signals, enabling labeling of non-repetitive regions with as few as four unique sgRNAs (Qin et al., 2017). However, challenges with this method arose as the binding motifs inserted into the 3’ end of the sgRNA scaffold were found to interfere with the stability of the sgRNA, resulting in lower labeling efficiency (Ma et al., 2018). This limitation was overcome in the CRISPR-Sirius system by inserting arrays of aptamers into the stem loop of the sgRNA scaffold. Another modification to the sgRNA involves incorporating arrays of fluorescent RNA aptamers to amplify the signal in the dCas13/sgRNA/FP system, enabling simple and sensitive imaging and tracking of endogenous RNA. (Tang et al., 2022).

A recent study reported a robust CRISPR-mediated fluorescence *in situ* hybridization amplifier (CRISPR FISHer) system, which exploited protein trimerization domain-mediated phase separation to assemble copies of foldon-FP-PCP and sgRNA-8× PP7 to visualize non-repetitive DNA loci using a single sgRNA (Lyu et al., 2022). The CRISPR FISHer system generated a significantly higher signal-to-background ratio than the CRISPR-Sirius system, indicating that it is the most sensitive labeling system with the highest signal-to-background ratio among the redesigned sgRNA systems. The multiple fluorescent proteins aggregated by the foldon-FP-PCPs and sgRNA-8× PP7s were responsible for the high sensitivity of the CRISPR FISHer system. Moreover, this system allowed for real-time monitoring of chromosomal dynamics, making it a powerful tool for investigating biological events. Another method called SIMBA utilizes dCas9-Suntag/scFv-FKBP/FRB-HP1α to achieve labeling of non-repetitive loci (Peng et al., 2022). The HP1α protein was used to induce assembly, similar to the function of foldon in CRISPR FISHer. However, the signal-to-background ratio of the SIMBA system has not been compared to other labeling systems. A CRISPR/Casilio-based imaging method was also developed recently for labeling non-repetitive genomic locus with only one sgRNA. The scaffold of the sgRNA contains 15 copies of PUF binding sites c (15× PBSc) (Clow et al., 2022). In the system, each sgRNA can recruit 15 fluorescent proteins, and the labeling specificity was evaluated by co-labeling with FISH probe. The CRISPR FISHer, SIMBA, and CRISPR/Casilio-based systems are the newest techniques reported for visualizing non-repetitive genomic regions using only one sgRNA, indicating that genomic imaging will be less laborious and more efficient for hard-to-access regions. These techniques enhance the brightness of each single DNA/RNA binding protein or sgRNA in the complex, similar to the strategy that uses a single fluorophore with intensive brightness.

### 4.2 Single fluorophore strategy of labeling elements

In the strategy of single fluorophore labeling, quantum dots (QDs) have been used for genome labeling in the TALE and CRISPR/dCas9 systems for targeting non-repetitive loci. QDs have ideal properties for single-molecule imaging due to their brightness and long lifetimes (Michalet et al., 2005; Resch-Genger et al., 2008; Wegner and Hildebrandt, 2015). For example, one TALE has been fused with one QD to label single genomic loci of HIV-1 proviral DNA sequences in live host cells. Two TALE-QDs were also colocalized to determine the locus of interest in the system (Ma et al., 2017a). Additionally, QDs have been coupled with dCas9 to label integrated HIV-sequences in living cells, which showed imaging ability of non-repetitive loci (Ma et al., 2017b). A molecular beacon (MB) has been shown to exhibit a fluorescence increase of up to 100-fold upon hybridization to target RNA. The Molecular beacons (MBs) have also been used for live cell imaging by coupling with the CRISPR/dCas9/sgRNA system. In this labeling method, the sgRNA was engineered to integrate a unique MB target sequence (sgRNA-MTS), and MBs were then delivered to hybridize to MTS to illuminate the target sequence (Wu et al., 2018). Telomeres were labeled and verified by co-labeling with EGFP-TRF1. The sgRNA-MTS was further modified to carry two distinct molecular beacons that can undergo fluorescence resonance energy transfer (FRET), which was called the CRISPR/dual-FRET molecular beacon system (Mao et al., 2019). When two fluorophores on the two MBs are in sufficiently close proximity, the excited-state energy of the donor molecule is transferred to the neighboring acceptor fluorophore whose fluorescence intensity was enhanced (Cardullo et al., 1988). The enhanced labeling system was also able to label non-repetitive genomic loci with only three unique sgRNAs. The signal amplification strategies that we discussed aim to enhance the fluorescence intensity at the target DNA or RNA locus, and researchers have employed multiple fluorophores and single fluorophores with superior brightness to achieve this goal. Excellent labeling systems have been constructed using either of these strategies, and among them, a single sgRNA is enough to label one non-repetitive genomic locus, making it the most sensitive method for DNA imaging to date. However, even with these signal amplification methods, the potentially high background fluorescence readout around the target loci introduced by the enhanced fluorescent intensity of the labeling system is still an issue that researchers need to pay attention to. The labeling elements are typically expressed through plasmid transfection in living cells, which means that the number of the labeling elements expressed can not only meet the demand of the target locus but also move freely around the locus. For example, the dCas9 protein or sgRNA with multiple fluorophores that are not tagged to the locus of interest may move freely in the cell nucleus, causing background fluorescence. When the locus of interest contains only a few or a single specific sequence that can be labeled by the complex of dCas9/sgRNA/multiple fluorophores, the fluorescence intensity of the target locus might be difficult to distinguish from that of the free complex around it. Therefore, researchers have also explored ways to decrease the background fluorescence to increase the signal-to-background (S/B) ratio, which we will discuss in the next section.

## 5 Background fluorescent signal reduction techniques

In signal amplification labeling systems, free background fluorescence can mainly be caused by the labeling elements that are not attached to the DNA or RNA loci and move freely in the nucleus. Therefore, the intensity and number of the labeling elements become the main factors that contribute to the background fluorescence. To reduce the background fluorescence, researchers have explored two strategies: decreasing the fluorescent intensity of the free labeling elements and decreasing the number of the free labeling elements (Table 1).

### 5.1 Fluorescent intensity reduction strategy of labeling elements

To decrease nuclear background fluorescence in modified MS2/PP7, TALE, CRISPR/dCas9-SunTag, and CRISPR/dCas13-SunTag systems, the bimolecular fluorescent complementation system (BiFC) has been used (Wu et al., 2014; Hu et al., 2017; Chen et al., 2022). These systems utilize the spatial proximity of two split non-fluorescent fragments (N-terminal fragments and C-terminal fragments) to complement each other and reform fluorescent proteins, reducing the background fluorescence as two free fragments cannot emit fluorescence. A BiFC-TALE system was constructed to label telomeres and centromeres in living cells. In this system, the C-terminal fragment and the N-terminal fragment from mVenus fluorescent protein were respectively fused to the C-terminus of a pair of TALE modules. The pair of modules were designed to bind head-to-head on different strands of the double-stranded DNA and the spacer length within the pair was <10 nm for the complementation of the two non-fluorescent fragments. By the background reduction strategy, a significantly improved fluorescent S/B ratio was reported in the system. In another SunTag-dCas9-MCP-BiFC system, scFv-Venus C-terminal (scFv-VC) and MCP-Venus N-terminal (MCP-VN) were constructed based on the dCas9-SunTag labeling system (Hong et al., 2018). In this system, multiple scFv-VCs and MCP-VNs approximate as the scFv-VCs were recruited to the GCN4 array in dCas9-SunTag and MCP-VNs were recruited to MS2 in the sgRNA scaffold. Then, the two fragments reform Venus to illuminate the target genomic loci. The signal-to-noise (S/N) ratio of this system was reported to be the highest among several BiFC systems and the original dCas9-SunTag system when labeling the endogenous human *MUC4* genomic locus containing 90 copies of repetitive sequence. This increased S/N ratio highlights the potential of background reduction methods. However, the BiFC system has limitations, including long maturation time and low protein recovery rate. In addition to the BiFC system, several studies have reported that sgRNA scaffolds with fluorescent signals can be degraded when not targeting the locus of interest, providing another choice to reduce the background fluorescence in labeling systems (Gu et al., 2018).

Fluorophores of fluorescent proteins are typically non-fluorescent in solution but become highly fluorescent when bound to a folded protein (Filonov et al., 2014). Thus, RNA aptamer systems like Spinach, Broccoli, Mango, and Peppers can also be categorized as fluorescent background reduction systems since these aptamers mimic the function of fluorescent proteins and switch on their non-fluorescent fluorophores (Su and Hammond, 2020; Zhou and Zhang, 2022). The fluorescence intensity of the fluorophores increases up to over 1,000 folds when binding with the specific RNA aptamers in live cells. In Peppers and Mango systems, a higher S/B ratio was achieved compared to the MCP/MS2 system, indicating that reducing the background fluorescence can increase the S/B ratio. An aptamer-initiated fluorescence complementation (AiFC) method for RNA imaging was developed by engineering Broccoli into two split fragments (Wang et al., 2018b). The split fragments can tandemly bind to endogenous mRNA and reform the fluorophore-binding site, allowing for visualization of the RNA molecules through fluorescence. The AiFC system has been successfully used to illuminate endogenous mRNA in live cells, providing researchers with a novel approach to reduce fluorescence background and optimize RNA labeling systems (Wang et al., 2018b).

### 5.2 Number reduction strategy of labeling elements

Recently, we reported two systems that decrease the number of background fluorescent proteins to increase the S/B ratio (Lu et al., 2021; Hou et al., 2022). In the reduced nuclear background system (RNBS), the scFv-sfGFP-NLS (signal module) in the dCas9-SunTag system was redesigned to increase its size and remove the nuclear entering guided signal NLS. As a result, most of the modified signal modules were limited to the cytoplasm by the nuclear pore complex, and only a few entered the nucleus for labeling. The S/B ratio was increased by 1.6-fold when labeling a low repetitive genomic locus with RNBS, and the system was shown to be robust and efficient for low-repetitive genomic loci. In the CRISPR-based light-inducible background reduction (CRISPR-LIBR) method, a light-inducible nuclear export tag (LEXY) was fused with the dCas9-SunTag system. The untargeted LEXY-tagged sfGFP were controllably transferred to the cytoplasm upon blue light irradiation, thereby reducing the number of fluorescent proteins inside the nucleus. The S/B ratio was also significantly increased after the irradiation, and seven randomly chosen genomic loci with low repeats (repetitive sequence) were efficiently labeled. Among the 7 loci, C3-18 (chromosome 3, 18 repeats), C7-15 (chromosome 7, 15 repeats) and C3-9 (chromosome 3, 9 repeats) were also selected for imaging by traditional dCas9-EGFP method, but C7-15 and C3-9 cannot be detected. The two systems demonstrated that decreasing the number of background fluorescent proteins can markedly increase the S/B ratio and offer a new approach for researchers to explore new labeling systems.

To improve RNA imaging, a nuclear localization sequence (NLS) has been added to MCP-FP and PCP-FP to guide labeling elements into the nucleus (Bertrand et al., 1998; Grunwald and Singer, 2010). This reduces the number of fluorescent proteins in the cytoplasm, decreasing background fluorescence when imaging RNA tagged with MS2 and PP7.

## 6 Application of the optimized labeling system

The ability to reveal the dynamic details of genomic three-dimensional (3D) organization and transcriptomic function in live cells relies on the toolbox discussed above. The following examples illustrate how DNA and RNA imaging systems, which employ signal amplification or background reduction strategies, can be used to investigate DNA repair, intrachromosomal interactions, RNA splicing and RNA-RNA interaction.

In one study, the CRISPR FISHer system was used to label the PPP1R2 gene, and the CRISPR-Sirius system was used to label an adjacent repeated genomic locus at Chr3q29 (∼500 repeats, termed Chr3Rep). The system was used to observe CRISPR-mediated DNA double-strand breaks (DSBs) and nonhomologous end joining (NHEJ) repair in chromosome 13. The dynamics of the two loci labeled with two fluorescent proteins were tracked before and after chromosomal breakage for 135 min. Intrachromosomal dissociation and rejoining were clearly shown during the process (Lyu et al., 2022). Another example is to investigate genomic organization by DNA imaging tools. A ∼500 kb loop domain between the IER5L promoter (IER5L-P) and its super-enhancer (IER5L-SE) was observed to be lost upon the depletion of RAD21 in a previous study (Rao et al., 2017). In an engineered HCT116 cell line with endogenous RAD21 fused to an auxin-inducible degron, the IER5L-P and IER5L-SE loci were labeled using a CRISPR/Casilio-based system (Clow et al., 2022). When treated with auxin, the spatial distance between the two loci significantly increased compared to the untreated control, demonstrating the involvement of RAD21 in loop domain formation and intrachromosomal interaction. The two applications of the DNA labeling systems exhibit the ability to visualize DNA loci dynamically in real time by tagging only a segment containing fewer than 30bp (the size of an sgRNA). The improved resolution in tagging genomic segments allows for a more detailed understanding of chromosomal behavior and provides more dynamic information about their biofunctions.

The dCas13a-SunTag-BiFC system was used to detect a premature termination codon (PTC) mutation-induced exon skipping (Chen et al., 2022). Exon 2 of the exogenous Oxt gene was targeted, and the exogenous natural Oxt gene was used as a control. The signal of exon 2 was separated from the signal of the Oxt gene for the mutation sample compared to that of the control, indicating a rapid detection of PTC mutation-induced exon skipping. In another study, a CRISPR-dPspCas13b system with fluorescent RNA aptamers in sgRNA (CasFAS) was used to image two RNA species in live cells. Broccoli and Pepper were respectively used in the dCas13/sgRNA complex for simultaneous detection of *NEAT1* and *MALAT1*. The colocalized signals were consistent with previous study, in which the 5′region of *NEAT1* interacts with *MALAT1* (Cai et al., 2020). RNA is subject to more dynamic behavior in live cells than DNA, and the development of these RNA tagging techniques would expand the range of observable RNA species. Combining RNA labeling systems with DNA labeling systems would provide a clearer understanding of how genomes initiate their genetic functions and achieve their goals. This would offer a more comprehensive view of cellular processes and their regulation.

## 7 Perspective

Imaging tools for DNA and RNA in live cells have been developed for years, and their roles in dynamically recording the behaviors of biomolecules have been key in proving their importance. This review specifically focuses on signal amplification and background reduction strategies, each with their own distinct advantages and considerations. In a labeling system, the effect of signal amplification and background reduction can counteract with each other. The presence of free fluorescent elements can cause background noise, but they can also act as targeting elements on the loci of interest, leading to increased fluorescence. Similarly, with a background reduction system, targeting elements that are meant to decrease fluorescence can inadvertently reduce target fluorescent signals. Therefore, newly designed labeling systems must consider these potential issues in order to be efficient and useful. With the advent of new tagging techniques, we believe that more optimal labeling systems will be developed in the future.
